# Molecular Characterization of Imported and Autochthonous Dengue in Northeastern Spain

**DOI:** 10.3390/v13101910

**Published:** 2021-09-23

**Authors:** Jessica Navero-Castillejos, Rosa Benitez, Nuria Torner, José Muñoz, Daniel Camprubí-Ferrer, Aida Peiró-Mestres, Elena Sulleiro, Aroa Silgado, Verónica Gonzalo, Teresa Falgueras, Izaskun Alejo-Cancho, Montserrat Roldán, Virginia Plasencia, Rosa Albarracin, Josefa Perez, Alexander Navarro, Ana Calderón, Rosa Rubio, Mireia Navarro, Miguel Micó, Jaume Llaberia, María Navarro, Josep Barrachina, Anna Vilamala, Carmina Martí, María Ángeles Pulido, María Paz Sanchez-Seco, Ana Vazquez, Ana Martínez, Mireia Jané, Miguel Julián Martínez

**Affiliations:** 1 Department of Clinical Microbiology, Hospital Clinic de Barcelona, 08036 Barcelona, Spain; jessica.navero@isglobal.org (J.N.-C.); aida.peiro@isglobal.org (A.P.-M.); vgonzal1@clinic.cat (V.G.); i.alejo.cancho@gmail.com (I.A.-C.); ralbarra@clinic.cat (R.A.); anavarr2@clinic.cat (A.N.); minavarr@clinic.cat (M.N.); barrachina@clinic.cat (J.B.); 2 Barcelona Institute for Global Health (ISGlobal), Hospital Clinic de Barcelona, Universitat de Barcelona, 08036 Barcelona, Spain; jose.munoz@isglobal.org (J.M.); daniel.camprubi@isglobal.org (D.C.-F.); mroldan@clinic.cat (M.R.); 3 North Metropolitan International Health Unit PROSICS, Hospital Universitari Germans Trias i Pujol, 08916 Badalona, Spain; rmbenitezd.germanstrias@gencat.cat; 4 CIBER Epidemiology and Public Health CIBERESP, University of Barcelona, 08036 Barcelona, Spain; nuriatorner@ub.edu; 5 Department of Microbiology, Vall d’Hebron University Hospital, PROSICS, 08035 Barcelona, Spain; esulleir@vhebron.net (E.S.); aroa.silgado@vhir.org (A.S.); 6 Hospital Municipal de Badalona, Badalona Serveis Assistencials, 08911 Badalona, Spain; tfalgueras@bsa.cat (T.F.); acalderon@bsa.cat (A.C.); 7 Microbiology Laboratory, Catlab, 08232 Viladecavalls, Spain; vplasencia@catlab.cat (V.P.); jperez@catlab.cat (J.P.); RRubio@catlab.cat (R.R.); 8 Microbiology Department, Xarxa Assistencial Universitària de Manresa, 08243 Manresa, Spain; mmico@althaia.cat; 9 Hospital de Barcelona, Societat Cooperativa d’Instal·lacions Assistencials Sanitàries (SCIAS), 08034 Barcelona, Spain; jllaberia@sciashdb.com; 10 Microbiology Department, Hospital Universitari de Vic, 08500 Barcelona, Spain; mnavarro@chv.cat (M.N.); avilamalab@chv.cat (A.V.); 11 Hospital General de Granollers, 08402 Granollers, Spain; cmarti@fphag.org (C.M.); apulido@fphag.org (M.Á.P.); 12 Centro Nacional de Microbiología, Instituto de Salud Carlos III, 28222 Madrid, Spain; paz.sanchez@isciii.es (M.P.S.-S.); a.vazquez@isciii.es (A.V.); 13 Centro de Investigación Biomédica en Red en Epidemiología y Salud Pública (CIBERESP), 28029 Madrid, Spain; 14 Public Health Agency of Catalonia, Generalitat of Catalonia, 08005 Barcelona, Spain; a.martinez@gencat.cat (A.M.); mireia.jane@gencat.cat (M.J.)

**Keywords:** dengue, molecular epidemiology, autochthonous transmission, surveillance

## Abstract

Dengue is the most significant arbovirus worldwide and a public health threat to non-endemic areas in which *Aedes* vectors are present. Autochthonous dengue transmission has been reported in several European countries in the last decade. Infected travelers from endemic regions arriving to areas colonized by *Aedes albopictus* in Europe need to be monitored in surveillance and control programs. We aimed to perform molecular characterization of RT-PCR-positive dengue cases detected in Catalonia, northeastern Spain, from 2013 to 2018. The basic demographic information and the geographical regions of importation were also analyzed. One-hundred four dengue cases were studied (103 imported infections and the first autochthonous case in our region). The dengue virus strains detected were serotyped and genotyped using molecular methods, and phylogenetic analyses were conducted. All four dengue serotypes were detected in travelers, including up to 10 different genotypes, reflecting the global circulation of dengue in endemic areas. The primary travel-related case of the 2018 autochthonous transmission was not identified, but the molecular analysis revealed dengue serotype 1, genotype I of Asian origin. Our results highlight the diversity of imported dengue virus strains and the role of molecular epidemiology in supporting arbovirus surveillance programs.

## 1. Introduction

*Dengue virus* (DENV) is an enveloped, single-stranded, positive-sense RNA arthropod-borne virus that belongs to the genus *Flavivirus*, family *Flaviviridae*. Dengue transmission occurs primarily through the bites of infected *Aedes* mosquitoes, with *Ae. aegypti* being the main vector worldwide [1]. Dengue is endemic in more than 120 countries through tropical and subtropical areas, and up to 390 million DENV infections may occur annually [2]. The disease can also be transmitted by *Ae. albopictus*, an efficient invasive species with high ecophysiological plasticity [3] that has spread around the globe in the recent decades [4]. The expansion of *Ae. albopictus* in the European region has led to local transmission of DENV in the recent years in France, Croatia, Spain and Italy [5]. The clinical spectrum of dengue varies from asymptomatic infections (which are frequent) to classical dengue fever and, in a minority of cases, a severe life-threatening infection. Dengue is clinically classified as i) dengue with or without warning signs or ii) severe dengue, which reflects the dynamic nature of the infection [1,6]. The clinical manifestations and severity of dengue depend on various factors such as the genetics of the virus and the host, with previous infection with a different DENV serotype being one of the major risk factors for severe disease [7]. Diagnosis is based on viral detection assays, mainly reverse transcription polymerase chain reaction (RT-PCR) and non-structural 1 (NS1) antigen detection assays during the acute viremic phase and serological tests for detection of IgM and IgG against DENV, which may show cross-reactions with antibodies against other flaviviruses [8].

Four genetically and antigenically distinct serotypes have been described (from DENV-1 to DENV-4), sharing around 65% amino acid homology. Within each serotype, multiple genotypes have been described, which typically show less than 6% nucleotide divergence [9,10,11]. Strains detected at different timepoints and in different geographical areas are described for each genotype as follows [12,13]: 

(1) Dengue serotype 1 (DENV-1) is divided into five genotypes (I–V): DENV-1 genotype I includes strains from Southeast Asia, China and East Africa; genotype II includes strains from Thailand (1950s–1960s); genotype III is represented by a sylvatic strain detected in Malaysia; genotype IV includes strains from the West Pacific islands and Australia. Recently, a sixth genotype (VI) has been proposed based on the divergence of a sylvatic strain imported from the rainforests of Brunei [14];

(2) Dengue serotype 2 (DENV-2) is divided into six genotypes: the American–Asian genotype (strains from Thailand, Vietnam and the Americas); the Asian I genotype (strains from Malaysia and Thailand); the Asian II genotype (strains from Vietnam, China, Taiwan, Sri Lanka and the Philippines); the American genotype (strains from Latin America as well as other strains from the Caribbean, the Indian subcontinent and the Pacific Islands collected in the 1950s and 1960s); the Cosmopolitan genotype (strains from Australia, Africa, the Pacific and Indian Ocean islands, the Indian subcontinent and the Middle East); and the Sylvatic genotype (strains from West Africa and Southeast Asia);

(3) Dengue serotype 3 (DENV-3) contains five genotypes. Genotype I includes strains from Indonesia, Malaysia, the Philippines and recent isolates from the South Pacific islands. Genotype II includes strains from Thailand, Vietnam and Bangladesh. Genotype III includes strains from Sri Lanka, India, Africa, Samoa and a 1962 strain from Thailand. Genotype IV includes strains from Puerto Rico, Latin and Central America and the 1965 Tahiti strain. Genotype V includes strains from the Philippines and the Americas; sylvatic DENV-3 strains have not been sequenced yet, but seroconversion of sentinel monkeys in Malaysia has been reported;

(4) Four genotypes have been described for dengue serotype 4 (DENV-4): genotype I (strains from Thailand, the Philippines, Sri Lanka and strains imported to Japan from Southeast Asia); genotype II (strains from Indonesia, Malaysia, Tahiti, the Caribbean and the Americas); genotype III (recent strains from Thailand that differ from other Thai isolates); and genotype IV (sylvatic strains from Malaysia).

Molecular epidemiology has greatly contributed to our understanding of the history, evolution and diversity of DENV [10,13]. In addition, it is an essential tool for the investigation of the origins of DENV outbreaks or introductions of the virus into nonendemic areas [15,16,17]. Around 200 imported dengue cases are reported yearly to the Spanish National Epidemiological Surveillance Network, and Catalonia is one of the regions in which more cases are detected [18]. Local transmission of dengue in Spain has been reported in 2018 for the first time and again in 2019. In 2018, six cases were reported in Murcia (Southeastern Spain) [19], and one case in Catalonia (Northeastern Spain). In 2019, one autochthonous case was detected again in Catalonia [20], and another case, sexually transmitted, in Madrid [21]. In this study, we aimed to perform genetic characterization of imported and autochthonous DENV strains detected in Northeastern Spain (Catalonia region) between 2013 and 2018.

## 2. Materials and Methods

### 2.1. Study Design

Dengue cases were detected by means of routine diagnosis of patients attending the participating hospitals through the public health arbovirus surveillance and control program of the Public Health Agency of Catalonia (Northeastern Spain). In this program, patients with an arbovirus-compatible febrile syndrome are screened for *dengue*, *chikungunya* and *Zika viruses* at two designated reference laboratories (Hospital Clinic de Barcelona, Barcelona, Spain, and Hospital Vall d’Hebron, Barcelona, Spain). Epidemiological surveillance and entomological control measures are conducted to reduce the risk of autochthonous transmission. We analyzed the acute dengue cases diagnosed by means of positive real-time RT-PCR between 2013 and 2018. Demographic and epidemiological data were retrospectively reviewed. The study was approved by the Clinical Research Ethics Committee of Hospital Clinic de Barcelona (files HCB/2018/0931, HCB/2021/0709). Informed consent for publication was obtained from the autochthonous case described in this manuscript.

### 2.2. Nucleic Acid Extraction, DENV Genome Detection and DENV Serotyping

Viral RNA was isolated from 500 µL of serum using an automated system (MagNA Pure Compact; Roche Diagnostics, Mannheim, Germany) with a MagNA Pure Compact Nucleic Acid Isolation Kit I—Large Volume. A commercial specific real-time RT-PCR assay (LightMix Modular Dengue, TIB Molbiol, Berlin, Germany) was used to amplify and detect the DENV genome in a LightCycler 480 II thermal cycler (Roche Diagnostics) with the RNA Process Control KIT master mix (Roche Diagnostics). Five microliters of purified nucleic acids were used in a 20 µL RT-PCR reaction following the manufacturer’s instructions. Serotyping of DENV-positive RT-PCR samples was performed by means of serotype-specific real-time RT-PCR using the Centers for Disease Control and Prevention (CDC) DENV-1–4 Real-Time RT-PCR Multiplex Assay on a Viasure 48 thermocycler (Certest Biothech, Zaragoza, Spain) or an in-house generic flavivirus RT-PCR assay followed by Sanger sequencing of the amplicons [22].

### 2.3. Genotyping of DENV and Phylogenetic Analysis

For genotyping of dengue viruses, regions of the viral genome for each DENV serotype were amplified by nested RT-PCR and sequenced by the Sanger method in an external company (Genewiz, Leipzig, Germany). Different sets of primers targeting the DENV envelope (*E*) gene were used for each serotype in order to amplify a genome fragment of around 1700 bp covering the complete or nearly complete *E* gene of each of the four DENV serotypes. For samples that failed to amplify the *E* gene due to lower viral loads, another protocol targeting a shorter fragment (around 500 bp) of the *E-NS1* junction of the viral genome was used. The primers used in this study were previously described/modified from the literature [23,24] or designed de novo. For the primer design, a total of 327 DENV reference sequences of the last 17 years were retrieved from the Dengue Virus Database of the National Center for Biotechnology Information (NCBI), (https://www.ncbi.nlm.nih.gov/genomes/VirusVariation/Database/nph-select.cgi?taxid=12637, accessed on 10 January 2017). These strains covered all of the described DENV genotypes and different geographical areas. The sequences of each serotype were aligned with the Molecular Evolutionary Genetics Analysis version 7 software [25] to identify conserved regions suitable for amplification. The viability and adequacy of the designed and modified primers were evaluated in silico through the Primer3 [26], PrimerMap [27] and Amplyfx software ( https://inp.univ-amu.fr/en/amplifx-manage-test-and-design-your-primers-for-pcr, Aix-Marseille Univ, CNRS, INP, Inst Neurophysiopathol, Marseille, France, accessed on 10 January 2017). The primers used for amplification of DENV strains are shown in Table 1 and the primers used for sequencing were previously described [24].

RT-PCR reactions were performed with a One-Step RT-PCR kit (Qiagen GmbH, Hilden, Germany) following the manufacturer’s instructions using 5 µL of isolated RNA and 20 pmol of each primer. The thermal cycler conditions used were as follows: a reverse transcription step of 50 °C for 30 min followed by 15 min of denaturation at 94 °C; 40 cycles of denaturation (94 °C, 30 s), primer annealing (50 °C, 1 min) and primer extension (72 °C, 2.5 min); final incubation at 72 °C for 10 min. The second amplification reaction (nested PCR) was performed using 1 µL of the initial amplification product with a Go Taq PCR kit (Promega, Madison, Wisconsin, WI, US) and 20 pmol of each primer. Nested PCR reactions were carried out using initial denaturation (94 °C, 2 min) and 40 cycles of denaturation (94 °C, 30 s), primer annealing (50 °C, 45s), primer extension (72 °C, 2.5 min) and final incubation at 72 °C for 5 min. Amplified PCR products were verified by electrophoresis in 1% agarose gel. Available sequences for each DENV serotype and genotype were selected in Nextstrain (https://nextstrain.org/, accessed on 1 April 2021), Virus Pathogen Resources (VIPR, https://www.viprbrc.org/brc/home.spg?decorator=vipr, accessed on 1 June 2021) and GenBank (https://www.ncbi.nlm.nih.gov/genbank/, accessed on 1 June 2021) for phylogenetic analysis. The sequences obtained for every strain were aligned by means of the MUSCLE method using the MEGA7 software program and assembled in a consensus sequence. Then, all the consensus sequences were aligned with the corresponding reference sequences of the same serotype with the MUSCLE tool. The best maximum likelihood model found was used to construct phylogenetic trees, and the reliability of the tree was evaluated using the bootstrap method (1000 replicates).

## 3. Results

### 3.1. Imported Dengue Cases

**Figure 1:**
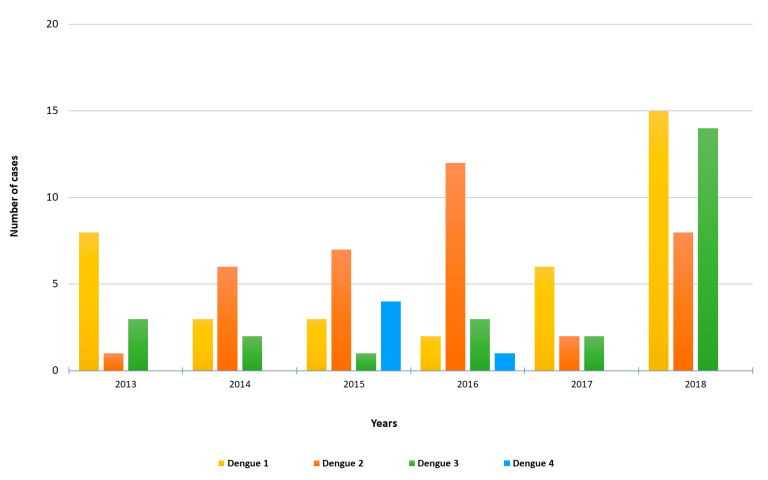
Number of imported RT-PCR-positive dengue cases by year and DENV serotype included in the study.

**Figure 2:**
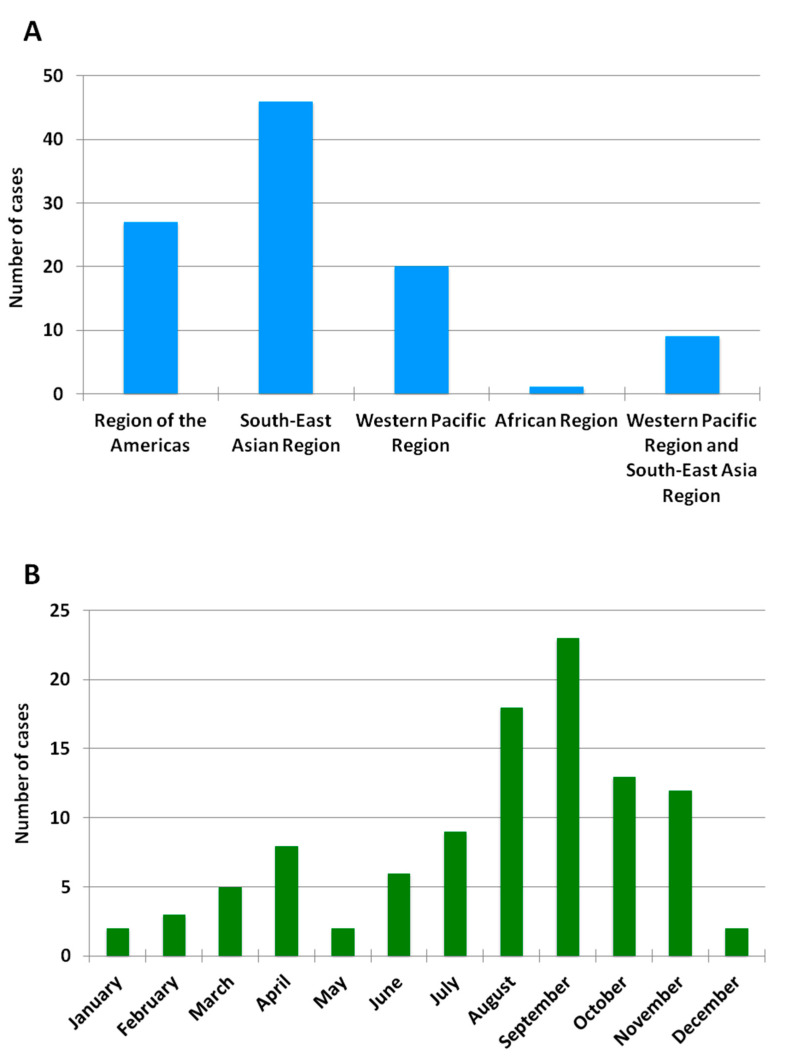
Geographic and monthly distribution of RT-PCR-positive imported dengue cases. (**A**) Number of imported RT-PCR-positive dengue cases by WHO region between 2013 and 2018. No cases were detected in travelers returning from the Eastern Mediterranean Region or the European Region. (**B**) Number of imported RT-PCR-positive dengue cases by month between 2013 and 2018.

A total of 103 cases of imported acute dengue infection diagnosed by a positive dengue RT-PCR between 2013 and 2018 were analyzed. The year in which more cases were detected was 2018 (approximately one third of the total number of cases), followed by 2016 and 2015 (Figure 1). The median age of the patients was 35 years (range, 19–72), and 57% were males. The WHO regions visited by the travelers where the dengue infections were acquired are represented in Figure 2A. Almost half (45%) of the dengue cases were imported from Southeast Asia, followed by the Americas (26%) and the Western Pacific (19%) Regions. Nine patients (9%) had visited two WHO regions during the trip (South-East Asian and the Western Pacific Regions). Only one viremic case was detected among travelers returning from Africa. No cases positive by RT-PCR were detected in travelers returning from the Eastern Mediterranean Region. The following countries were visited by the travelers diagnosed with dengue: Argentina, Brazil, Cambodia, Colombia, Costa Rica, Cuba, Dominican Republic, El Salvador, French Polynesia, Guatemala, India, Indonesia, Japan, Laos, Malaysia, Maldives, Myanmar, Mexico, Nepal, the Philippines, Singapore, Sri Lanka, Tanzania, Thailand, Uruguay, Venezuela and Vietnam. Fifteen patients visited more than one country during the trip. The distribution of viremic cases by month is shown in Figure 2B. The highest number of dengue cases was detected in September, and more than half of the acute dengue cases were diagnosed between August and October.

### 3.2. Molecular Characterization of Imported Dengue

Molecular serotyping of the 103 imported DENV strains revealed 37 cases of DENV-1, 36 cases of DENV-2, 25 cases of DENV-3 and 5 cases of DENV-4. The distribution of DENV serotypes diagnosed by year is shown in Figure 1. DENV-1 was the serotype most frequently observed in 2013, 2017 and 2018, whereas DENV-2 was the main serotype detected in 2014, 2015 and 2016. DENV-3 was mainly imported in 2018 (14 cases) by travelers returning from the South-East Asian and the Western Pacific Regions. Four dengue serotypes were detected in travelers returning from the South-East Asian, the Americas and the Western Pacific Regions. Moreover, all the four serotypes were imported from countries with widespread DENV circulation such as Thailand or the Philippines, and three different serotypes were imported from countries like India, Indonesia or the Dominican Republic.

**Figure 3:**
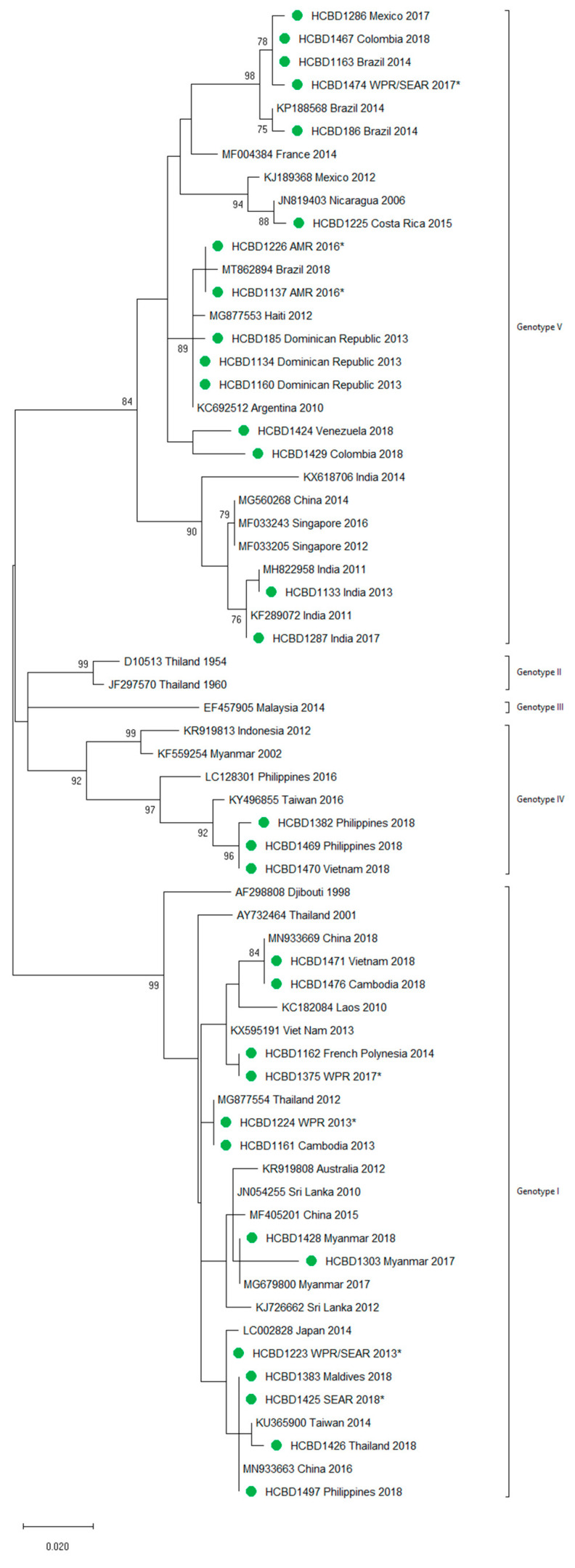
Phylogenetic tree of DENV-1 strains based on the *E/NS1* junction region. Strains are denoted by the GenBank accession number, place and year of isolation. The green dots indicate the strains sequenced in this study and the scale bar indicates substitutions per site. The analysis was performed using the maximum likelihood method (K2 + G) with a bootstrap of 1000 replicates.

**Figure 4:**
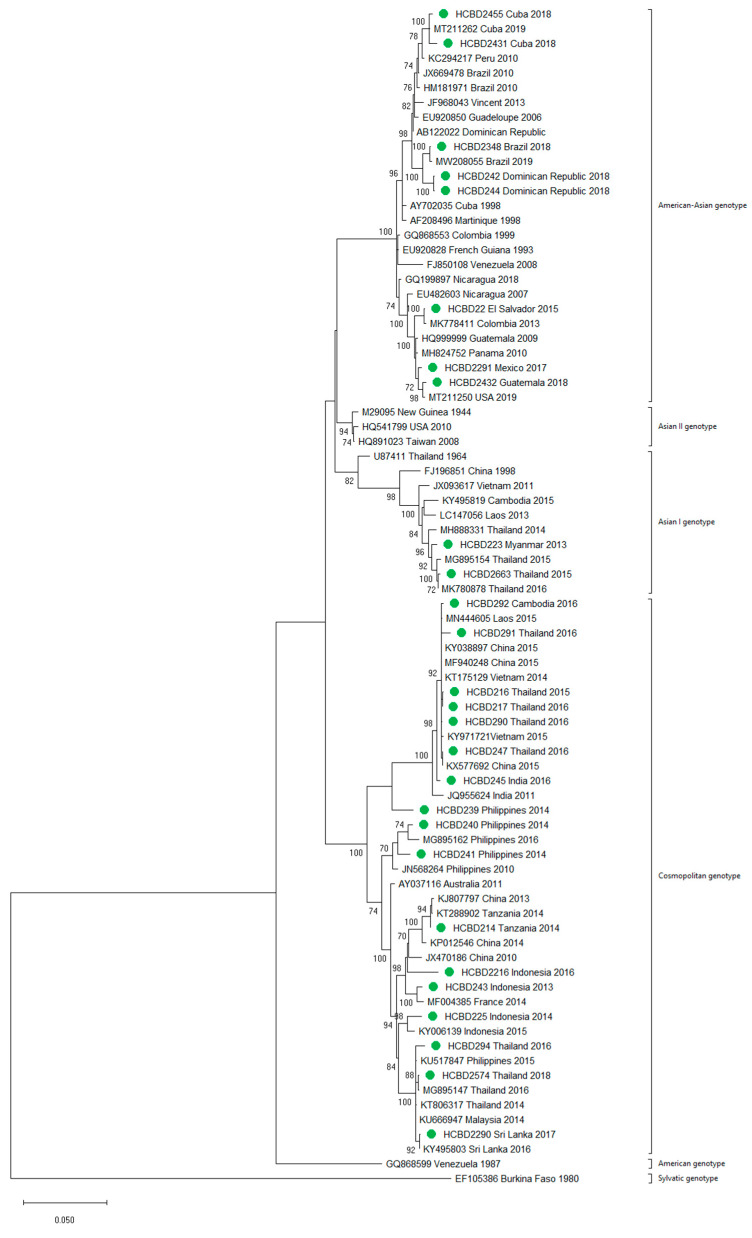
Phylogenetic tree of DENV-2 strains based on the *E* gene. Strains are denoted by the GenBank accession number, place and year of isolation. The green dots indicate the strains sequenced in this study and the scale bar indicates substitutions per site. The analysis was performed using the maximum likelihood method (TN93 + G + I) with a bootstrap of 1000 replicates.

**Figure 5:**
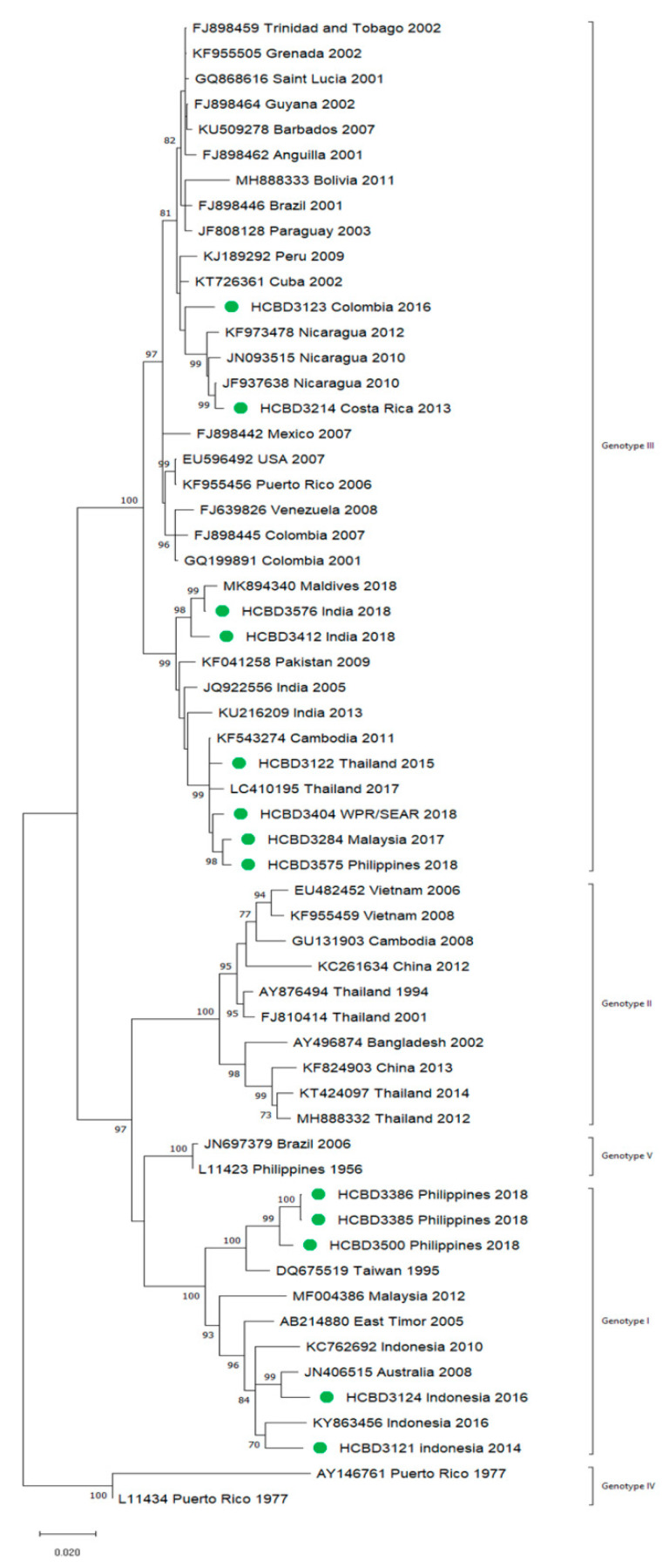
Phylogenetic tree of DENV-3 strains based on the *E* gene. Strains are denoted by the GenBank accession number, place and year of isolation. The green dots indicate the strains sequenced in this study and the scale bar indicates substitutions per site. The analysis was performed using the maximum likelihood method (TN93 + G + I) with a bootstrap of 1000 replicates.

**Figure 6:**
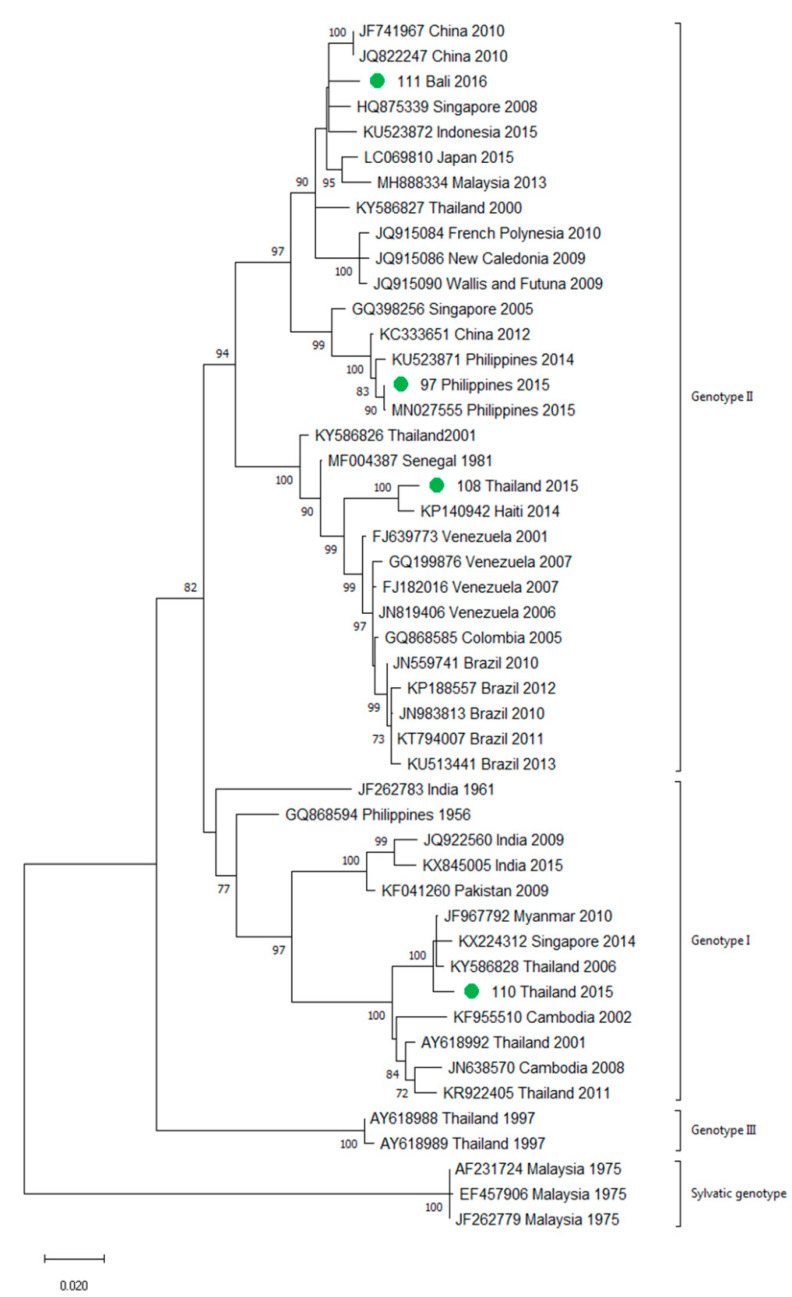
Phylogenetic tree of DENV-4 strains based on the *E* gene. Strains are denoted by the GenBank accession number, place and year of isolation. The green dots indicate the strains sequenced in this study and the scale bar indicates substitutions per site. The analysis was performed using the maximum likelihood method (TN93 + G + I) with a bootstrap of 1000 replicates.

A total of 90 imported dengue viruses could be sequenced for genotyping and phylogenetic analysis (34 DENV-1, 33 DENV-2, 18 DENV-3 and 5 DENV-4 cases). The GenBank accession numbers of the strains sequenced in this study are available in Table S1 (Supplementary Materials). Phylogenetic trees for each of the DENV serotypes were constructed with the strains sequenced in this study and other reference strains available in the GenBank and VIPR databases (Figure 3, Figure 4, Figure 5 and Figure 6). The complete or nearly complete sequence of the *E* gene was used for the construction of DENV-2, -3 and -4 phylogenetic trees. For DENV-1, as the complete *E* could not be amplified for a number of samples (13), we used the *E/NS1* junction region of the viral genome, which is also widely used for DENV molecular analysis [23].

A variety of DENV genotypes was detected in the period of study. For DENV-1 and DENV-2, up to three different genotypes were detected, whereas for DENV-3 and DENV-4, two genotypes were identified. The number of cases of specific genotypes for each DENV serotype is shown in Table 2. The DENV-2 Cosmopolitan genotype and the DENV-1 genotypes V and I were the genotypes more frequently recognized (21, 16 and 15 cases, respectively), accounting for 58.7% of the cases. Two distinct genotypes of the same DENV serotype could be identified in different travelers that had visited, for example, the Philippines (DENV-1 genotypes I and IV), Thailand (DENV-2 genotypes Asian I and Cosmopolitan), Indonesia (DENV-3 genotypes I and III) or Thailand (DENV-4 genotypes I and II).

In general, the sequenced strains detected grouped in the phylogenetic trees with other circulating viruses described in the areas visited by the patients. Travelers returning from Southeast Asia imported DENV-1 genotypes I and V and travelers visiting the Western Pacific Region were infected with DENV-1 genotypes I, IV and V. From the Americas, the only DENV-1 genotype detected was genotype V. For DENV-2, the Cosmopolitan genotype was the most frequently detected one; it was imported from all the regions except for the Americas. In the Americas, all the DENV-2 strains belonged to the American–Asian genotype, including two infections acquired in Cuba in 2018, which grouped with strains imported from Cuba to the USA that led to local dengue transmission in Florida [28]. The DENV-2 infection imported from Africa (Tanzania) belonged to the Cosmopolitan genotype, and the phylogenetic analysis revealed a strong identity with the strains isolated during the 2014 outbreak in Tanzania, which, in turn, are closely related with DENV-2 Asian strains. The DENV-2 Asian I genotype was identified only after trips to the South-East Asian Region. The DENV-2 analysis also included one strain that was detected after a trip to El Salvador and in a mosquito pool collected in the traveler’s garden in 2015 [29]. Interestingly, some strains of DENV-1 and DENV-2 imported from Brazil in 2016 and 2018 clustered with some recently described epidemic strains in this country (strains MW208055 and MT862894, Figure 3 and Figure 4). DENV-3 was acquired in the Americas (genotype III), South-East Asian and Western Pacific Regions (genotypes I and III from both areas). Four of the five DENV-4 strains detected were imported in 2015, a period in which DENV-4 emerged in some countries like the Philippines [30]. Genotype I was detected in one patient that had visited Southeast Asia, and genotypes I and II were identified in travelers returning from Southeast Asia and the Western Pacific.

### 3.3. First Autochthonous Case in Northeastern Spain

**Figure 7:**
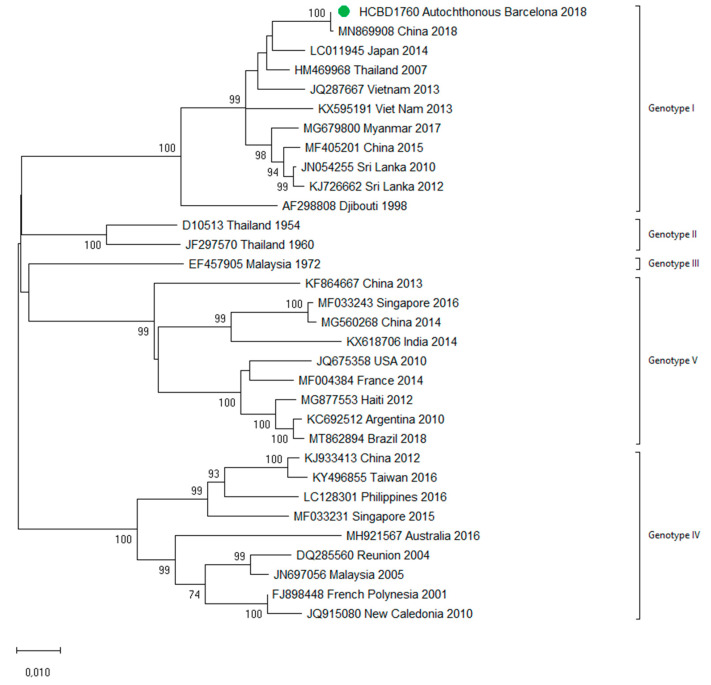
Phylogenetic analysis of the locally transmitted DENV-1 strain based on complete *E* and *NS1* genes. Strains are denoted by the GenBank accession number, place and year of isolation. The green dot indicates the strain of the autochthonous case and the scale bar indicates substitutions per site. The analysis was performed using the maximum likelihood method (TN93 + I) with a bootstrap of 1000 replicates.

In October 2018, a male in his twenties in the Province of Barcelona sought medical attention with headache and weakness. General hematological and biochemical blood tests were normal, and the patient was discharged with symptomatic treatment. Five days later, he presented with fever (38 °C) and nausea, and two days later with rash. A paranasal sinus radiography was normal. Blood tests showed slight leucopenia and thrombocytopenia, and he was diagnosed with a probable viral infection, with follow-up at the internal medicine department. The next day, he sought medical attention for persistence of fever and gastrointestinal symptoms, as well as progression of the rash. Blood tests showed leucopenia (2350/µL) and thrombocytopenia (83,000/µL). Tests for other viral infections such as *cytomegalovirus*, *Epstein–Barr virus* and *human immunodeficiency virus* were negative. Weakly positive IgM against *parvovirus B19* were detected, but the patient tested negative in the follow-up samples. A dengue virus infection was suspected during the last visit, and serological tests for DENV were IgM-positive and IgG-negative. As the patient had no recent history of traveling, a probable autochthonous dengue case was considered, and retrospective acute samples as well as follow-up samples were obtained. Confirmation of the diagnosis was obtained by detection of DENV RNA and DENV NS1 antigen in the stored samples from the first days of illness. Seroconversion of IgG was also detected in the follow-up samples. This represented the first case of locally transmitted dengue in Northeastern Spain. No other autochthonous cases were detected. DENV-1 genotype I was identified using the molecular analysis and no analogous DENV-1 strain was detected among the imported cases analyzed in this study, in agreement with the public health surveillance data that had not detected any epidemiologically linked cases in travelers that could represent the source of the transmission. The complete sequences of the *E* and *NS1* genes were amplified and used for further phylogenetic analysis (Figure 7). The strain clustered with other described DENV-1 Asian genotype I viruses. The patient featured complete resolution of symptoms and remained asymptomatic throughout the follow-up medical visits. Since autochthonous dengue was also detected in Murcia (Southeastern Spain) in August 2018, an interim analysis was conducted with the Spanish National Center for Microbiology comparing both strains (from Murcia and from Barcelona). The two strains grouped differently with other Asian strains, and therefore two different introductions of dengue in Spain leading to local transmission were concluded (data not shown).

## 4. Discussion

The global incidence of DENV infections has dramatically increased in the recent decades, putting almost half of the world’s population at risk of contracting dengue [1]. Although most DENV infections are asymptomatic or induce a mild disease, given the millions of cases that occur annually, severe dengue syndromes represent a major global health and economic problem [31]. The expansion of competent vectors for dengue is a public health concern for several countries of the European region. The unstoppable spread of *Ae. albopictus* in countries like Italy, France and Spain in the last 20 years [32] together with international travel have led to several outbreaks and cases of locally transmitted arboviral diseases transmitted by the tiger mosquito, such as dengue, chikungunya and Zika [5,33,34,35]. Vector competence studies on the European *Ae. albopictus* strains have shown that local mosquitoes are more susceptible to CHIKV than to DENV [36]. This may contribute to explaining why large outbreaks of CHIKV have occurred in countries like Italy despite the fact that the number of imported CHIKV infections is, by far, much lower than the number of imported DENV cases in Europe [37,38]. However, autochthonous transmission of DENV in the last decade in continental Europe has been reported almost every year since 2013, with several countries experiencing different local transmission events in the same year [5]. This underlines the need for reinforced surveillance and control programs in countries at risk of introduction of DENV. Few European series address in detail molecular characterization of imported DENV infections. In studies from 2002–2008 [24] and 2012–2014 [39], 186 and 141 strains were characterized, respectively. All DENV serotypes and multiple genotypes were detected in these studies, highlighting the diversity of imported infections and the role of travelers as sentinels of global DENV circulation. Our results are in agreement with these findings and provide a detailed description of DENV infections detected in Northeastern Spain, a region where *Ae. albopictus* has been well-established since 2004.

Monitoring and characterization of the detected DENV strains is relevant at different levels. Firstly, they allow for a better understanding of the diversity of imported infections, which might be relevant for risk assessment, as differences in vector competence, disease severity or cross-protective antibodies have been reported for distinct viral serotypes and strains [36]. We observed a significant diversity of DENV importations from different geographical areas, including all DENV serotypes and multiple genotypes. Secondly, they can shed light on local transmission events. In the autochthonous case studied, in the absence of a primary travel-related case identified, the molecular analysis revealed the Asian origin of the dengue introduction in our region. This case also highlights the need to increase clinical awareness due to the challenges of the clinical suspicion of autochthonous arboviral infections. Imported DENV cases undetected due to the lack of specificity of clinical presentation or asymptomatic dengue infections [40] may be the source of local transmission events, highlighting the difficulties of implementing surveillance programs for the prevention of DENV introduction. Third, the usefulness of analyzing DENV in travelers as a reflection of viral circulation in endemic regions is also underlined in our study. We detected DENV-1 and DENV-2 strains closely related to epidemic strains from Brazil, which were recently reported to silently circulate until appropriate conditions for emergence take place [41]. We also characterized strains imported from Cuba, where little information on dengue is available in the literature, and a strain imported from Tanzania.

In a large study on more than 40,000 travelers, dengue represented the second cause of febrile syndrome in travelers and the leading cause of fever in travelers returning from Southeast Asia, Latin America and the Caribbean [42]. We did not assess causes of fever in the travelers, and our numbers for DENV infections are only based on RT-PCR-positive samples. Many other imported DENV infections are diagnosed using serological methods or might be underdiagnosed. Thus, the viremic infections in our study are only a portion of the total number of imported DENV infections, but it is reasonable to assume that they are representative in terms of geographical areas of importation and months of detection. Of note, most of the infections in our study were diagnosed in the periods of *Ae. albopictus* activity in our region (usually from May to November) [43].

The effect of COVID-19 on dengue and other arboviral diseases might be uncertain. While the effects of lockdowns might be clear on imported DENV infections due to the decrease in trips, the impact of COVID-19 in DENV endemic areas is less certain. While some countries have reported a decline in cases, which may be influenced by a decline in the reporting of cases, other countries have reported record numbers of DENV infections [41,42]. Several authors have warned about the potential negative effects of the COVID-19 pandemic on the surveillance and control activities for arboviruses and that this may lead to a resurgence of arboviral diseases in the near future [42,43,44]. Enhancement of preparedness, strengthening of surveillance programs and systematic characterization of detected strains are needed in the fight against DENV introduction into nonendemic areas.

## Figures and Tables

**Table 1:** Primers used in this study for amplification of the *E* gene and the *NS1/E* junction. For each DENV serotype, forward (F) and reverse (R) primers, their use in the first round RT-PCR or in the nested PCR and the reference are indicated.

			Primer		Sequence (5′-3′)	Localization	Ref.
Envelope gene	DENV-1	RT-PCR	F	EGENE1-S	CCGAAACGTGGATGTCCTCTGARGG	756–780	[24]
			R	EGENE-R	TCCTCCCATGCCTTCCCRATGG	2553–2574	
		Nested PCR	F	F Nested	ATAGGAACATCCATYACYCAG	866–887	Designed de novo
			R	EGENE/NS-RR	TGRAAYTTRTAYTGYTCTGTCC	2502–2523	[24]
	DENV-2	RT-PCR	F	EGENE2-S	CTGAAACATGGATGTCATCAGAAGG	758–782	[24]
			R	RRT 2	GCYGARGCYARYTTTGARGGRG	2533-2555	Modified from [24]
		Nested PCR	F	F Nested	ATGGCRGCDATYYTGGCDYAY	844–865	Designed de novo
			R	R Nested	CGKGARTTCATYCCTATCCATGT	2348–2371	Designed de novo
	DENV-3	RT-PCR	F	EGENE3-S	CTCAAACCTGGATGTCGGCTGARGG	756–780	[24]
			R	RRT 3	ATYCCRCAVACTCCATTYTYCC	2561–2583	Modified from [24]
		Nested PCR	F	F NESTED	ATGYTGGTCACYCCATCCATG	911–932	Designed de novo
			R	R NESTED	TTGTAYTGYTCTGTCCARGTRTG	2511–2534	Designed de novo
	DENV-4	RT-PCR	F	EGENE4-S	CTGAGACATGGATGTCATCGGAAGG	760–784	[24]
			R	RRT 4	CACAGACCCCHTCTTTGTGRGC	2567–2589	Modified from [24]
		Nested PCR	F	F NESTED	TACTCAGRAABCCAGGATTYGC	869–890	Designed de novo
			R	R NESTED	YTCCATGACACYRCACAACCC	2478–2470	Designed de novo
E/NS1 junction	DENV-1	RT-PCR	F	FRTC 1	TGSYTGAGACYCARCAYGGNAC	1869–1890	Modified from [23]
			R	RRTC 1	YTCRTTTGATATYTGYYTCCAC	2620–2641	
		Nested PCR	F	FNC 1	GRAAATGTTYGARGCHACYGCCC	2130–2153	Modified from [24]
			R	RNC 1	TCYTCCCAYGCYYTYCCRATGG	2553–2574	
	DENV-2	RT-PCR	F	FRTC 2	TAGCWRRRACRCARCATGGAAC	1871–1889	Modified from [23]
			R	RRTC 2	CAGTTCYGGWGYTATYTGYYTCCAC	2622–2646	
		Nested PCR	F	FNC 2	CARTYARYATAGAAGCAGARCC	2027–2048	Modified from [24]
			R	RNC 2	GCYGAWGCYARYTTTGRRGGRG	2534–2555	
	DENV-3	RT-PCR	F	FRTC 3	TYTCHGARACRCARCAYGGRAC	1863–1884	Modified from [23]
			R	RRTC 3	BARYTCATTRGCTAYTTGCTTCCAY	2614–2638	
		Nested PCR	F	FNC 3	GRRAARATGTTYGAGRCSMCYG	2125–2146	Modified from [24]
			R	RNC 3	ATYCCRCAVACTCCATTYTYCC	2562–2583	
	DENV-4	RT-PCR	F	FRTC 4	TGGCAGAAACACARCATGGRAC	1873–1894	Modified from [23]
			R	RRTC 4	YARYTCRTTRGTTATTTGYTTCCAC	2624–2648	
		Nested PCR	F	FNC4	ATYGGYAAGATGTTYGAGTCY	2130–2150	Modified from [24]
			R	RNC 4	CACAGACCCCHTCTTTGTGRGC	2568–2589	

**Table 2:** DENV genotypes detected in travelers.

Serotype	Genotypes Detected	Number of Cases
DENV-1	I	15
	IV	3
	V	16
DENV-2	Asian I	3
	American/Asian	9
	Cosmopolitan	21
DENV-3	I	8
	III	10
DENV-4	I	1
	III	4

## Data Availability

All relevant data are within the paper. All the DENV sequences obtained in this study can be accessed at GenBank Table S1 (Supplementary Materials).

## Supplementary Materials

The following are available online at https://www.mdpi.com/article/10.3390/v13101910/s1, Table S1: title.
