# Supplementary material for: Molecular Characterization of Imported and Autochthonous Dengue in Northeastern Spain
Source: Viruses. 2021 Sep 23;13(10):1910. doi: 10.3390/v13101910 (PMC8539074; doi:10.3390/v13101910)
Supplement: Supplementary file 1 [file viruses-13-01910-s001.zip › viruses-1347968-supplementary.pdf]

Table S1. DENV sequences obtained in this study.

Strain	Serotype	Genotype	Year	Country	GenBank ID
HCBD1161	1	I	2013	Cambodia	MZ520678
HCBD1162	1	I	2014	French Polynesia	MZ520679
HCBD13N	1	I	2015	Sri Lanka	MZ520688
HCBD1303	1	I	2017	Myanmar	MZ520684
HCBD1375	1	I	2017	French Polynesia	MZ520685
HCBD1383	1	I	2018	Maldives	MZ520687
HCBD1426	1	I	2018	Thailand	MZ520690
HCBD1428	1	I	2018	Myanmar	MZ520691
HCBD1471	1	I	2018	Vietnam	MZ520696
HCBD1476	1	I	2018	Cambodia	MZ520697
HCBD1497	1	I	2018	The Philippines	MZ520698
HCBD1224	1	I	2013	WPR*	MZ519975
HCBD1223	1	I	2013	WPR/SEAR*	MZ519976
HCBD1425	1	I	2018	SEAR*	MZ519972
HCBD1559	1	I	2018	Vietnam	MZ520699
HCBD1760	1	I	2018	Spain	MZ613343
HCBD1382	1	IV	2018	The Philippines	MZ520686
HCBD1470	1	IV	2018	Vietnam	MZ520694
HCBD1469	1	IV	2018	The Philippines	MZ520695
HCBD1133	1	V	2013	India	MZ520675
HCBD1134	1	V	2013	Dominican Republic	MZ520676
HCBD1160	1	V	2013	Dominican Republic	MZ520677
HCBD185	1	V	2013	Dominican Republic	MZ520701
HCBD1163	1	V	2014	Brazil	MZ520680
HCBD186	1	V	2014	Brazil	MZ520702
HCBD1225	1	V	2015	Costa Rica	MZ520681
HCBD1660	1	V	2015	Costa Rica	MZ520700
HCBD1226	1	V	2016	AMR*	MZ519973
HCBD1137	1	V	2016	AMR*	MZ519974
HCBD1474	1	V	2017	WPR/SEAR*	MZ519971
HCBD1286	1	V	2017	Mexico	MZ520682
HCBD1287	1	V	2017	India	MZ520683
HCBD1424	1	V	2018	Venezuela	MZ520689
HCBD1429	1	V	2018	Colombia	MZ520692
HCBD1467	1	V	2018	Colombia	MZ520693
HCBD22	2	American-Asian	2015	El Salvador	MH253297
HCBD242	2	American-Asian	2016	Dominican Republic	MZ520920
HCBD244	2	American-Asian	2016	Dominican Republic	MZ520923
HCBD2348	2	American-Asian	2018	Brazil	MZ520915
HCBD2432	2	American-Asian	2018	Guatemala	MZ520922
HCBD2455	2	American-Asian	2018	Cuba	MZ520925
HCBD2431	2	American-Asian	2018	Cuba	MZ540899
HCBD2434	2	American-Asian	2018	Cuba	MZ540900
HCBD223	2	Asian I	2013	Myanmar	MZ520909
HCBD229	2	Asian I	2014	Thailand	MZ520912
HCBD2663	2	Asian I	2015	Thailand	MZ520927

HCBD214	2	Cosmopolitan	2014	Tanzania	MZ520905
HCBD225	2	Cosmopolitan	2014	Indonesia	MZ520910
HCBD228	2	Cosmopolitan	2014	Thailand	MZ520911
HCBD239	2	Cosmopolitan	2014	The Philippines	MZ520917
HCBD240	2	Cosmopolitan	2014	The Philippines	MZ520918
HCBD216	2	Cosmopolitan	2015	Thailand	MZ520906
HCBD217	2	Cosmopolitan	2015	Thailand	MZ520907
HCBD241	2	Cosmopolitan	2015	The Philippines	MZ520919
HCBD292	2	Cosmopolitan	2016	WPS/SEAR*	MZ520050
HCBD2106	2	Cosmopolitan	2016	Thailand	MZ520904
HCBD2216	2	Cosmopolitan	2016	Indonesia	MZ520908
HCBD243	2	Cosmopolitan	2016	Indonesia	MZ520921
HCBD245	2	Cosmopolitan	2016	India	MZ520924
HCBD247	2	Cosmopolitan	2016	Thailand	MZ520926
HCBD289	2	Cosmopolitan	2016	Indonesia	MZ520928
HCBD290	2	Cosmopolitan	2016	Thailand	MZ520929
HCBD291	2	Cosmopolitan	2016	Thailand	MZ520930
HCBD294	2	Cosmopolitan	2016	Thailand	MZ520931
HCBD2290	2	Cosmopolitan	2017	Sri Lanka	MZ520913
HCBD2291	2	Cosmopolitan	2017	Mexico	MZ520914
HCBD2574	2	Cosmopolitan	2018	WPR/SEAR*	MZ520051
HCBD2353	2	Cosmopolitan	2018	Thailand	MZ520916
HCBD3121	3	I	2014	Indonesia	MZ505710
HCBD3145	3	I	2014	WPR/SEAR*	MZ520119
HCBD3124	3	I	2016	Indonesia	MZ505713
HCBD3147	3	I	2016	Indonesia	MZ505724
HCBD3385	3	I	2018	The Philippines	MZ505716
HCBD3386	3	I	2018	The Philippines	MZ505717
HCBD3500	3	I	2018	The Philippines	MZ505719
HCBD3588	3	I	2018	Indonesia	MZ505723
HCBD3214	3	III	2013	Costa Rica	MZ505714
HCBD3260	3	III	2013	WPR/SEAR*	MZ520120
HCBD3122	3	III	2015	Thailand	MZ505711
HCBD3123	3	III	2016	Colombia	MZ505712
HCBD3284	3	III	2017	Malaysia	MZ505715
HCBD3412	3	III	2018	India	MZ505718
HCBD3575	3	III	2018	The Philippines	MZ505720
HCBD3576	3	III	2018	India	MZ505721
HCBD3586	3	III	2018	Guatemala	MZ505722
HCBD3404	3	III	2018	WPR/SEAR*	MZ520118
HCBD4110	4	I	2015	Thailand	MZ505612
HCBD4108	4	II	2015	Thailand	MZ505611
HCBD497	4	II	2015	The Philippines	MZ505614
HCBD4148	4	II	2015	Dominican Republic	MZ505615
HCBD4111	4	II	2016	Indonesia	MZ505613

*: For cases in which the country of acquisition dengue infection could not be ascertained, the WHO region/s visited is indicated. AMR: Region of the Americas, WPR: Western Pacific Region, SEAR: South-East Asia Region
